# The Roles of *ATP13A2* Gene Mutations Leading to Abnormal Aggregation of α-Synuclein in Parkinson’s Disease

**DOI:** 10.3389/fncel.2022.927682

**Published:** 2022-07-06

**Authors:** Fan Zhang, Zhiwei Wu, Fei Long, Jieqiong Tan, Ni Gong, Xiaorong Li, Changwei Lin

**Affiliations:** ^1^Department of Gastrointestinal Surgery, The Third Xiangya Hospital of Central South University, Changsha, China; ^2^Hunan Key Laboratory of Medical Genetics, School of Life Sciences, Central South University, Changsha, China; ^3^Key Laboratory of Molecular Precision Medicine of Hunan Province, Center for Medical Genetics, Institute of Molecular Precision Medicine, Xiangya Hospital of Central South University, Changsha, China

**Keywords:** *ATP13A2*, α-synuclein, Parkinson’s disease, autophagy, lysosome

## Abstract

Parkinson’s disease (PD) is the second most common neurodegenerative disease. PARK9 (also known as ATP13A2) is recognized as one of the key genes that cause PD, and a mutation in this gene was first discovered in a rare case of PD in an adolescent. Lewy bodies (LBs) formed by abnormal aggregation of α-synuclein, which is encoded by the SNCA gene, are one of the pathological diagnostic criteria for PD. LBs are also recognized as one of the most important features of PD pathogenesis. In this article, we first summarize the types of mutations in the ATP13A2 gene and their effects on ATP13A2 mRNA and protein structure; then, we discuss lysosomal autophagy inhibition and the molecular mechanism of abnormal α-synuclein accumulation caused by decreased levels and dysfunction of the ATP13A2 protein in lysosomes. Finally, this article provides a new direction for future research on the pathogenesis and therapeutic targets for ATP13A2 gene-related PD from the perspective of ATP13A2 gene mutations and abnormal aggregation of α-synuclein.

## Introduction

Parkinson’s disease (PD) is a common neurological disease that is caused by diseasemultiple factors, and its incidence increases with age (Pringsheim et al., 2014). According to the involvement of different genes, PD can be divided into autosomal dominant forms (PD caused by mutations in SNCA, PARK8, PARK17, PARK21, PARK22, and other genes) and autosomal recessive genetic forms (PD caused by mutations in PARK2, PARK6, PARK7, PARK9, PARK14, PARK15, PARK19, PARK20, PARK23, and other genes) (Schneider and Alcalay, 2017). The PARK9 gene (also known as ATP13A2) is located on chromosome 1p36, and it encodes a lysosome-related transmembrane P5 ATP transport enzyme that can transport inorganic cations and other substrates across the cell membrane (Inzelberg et al., 2018). An autosomal recessive mutation in this gene was first discovered in a Chilean family in 2006. Members of this family present a rare, juvenile-onset PD called Kufor-Rakeb syndrome (KRS) (Ramirez et al., 2006). This syndrome is generally seen in adolescents (< 20 years old), and patients present with motor dysfunction, including muscle stiffness (Paisán-Ruiz et al., 2010), learning disability (Behrens et al., 2010), fine motor dysfunction (Malakouti-Nejad et al., 2014), or behavior disorders (Schneider et al., 2010).

Although the etiology of PD may be multifactorial, the abnormal accumulation of α-synuclein is recognized as the key pathogenesis of the disease (Rocha et al., 2018). α-synuclein is a protein that is highly enriched in neurons and is mainly concentrated in presynaptic terminals (Ross et al., 2008). The presence of Lewy bodies (LBs) containing α-synuclein is a neuropathological sign of PD (Alvarez-Erviti et al., 2011). PD patients first develop abnormal α-synuclein aggregates, which further develop into neuronal inclusions called LBs or Lewy neurites (LNs) (Kalia and Lang, 2015). These aggregates affect the function and survival of neurons by disrupting important cellular processes (Roberts et al., 2015). The pathological manifestation is early progressive death of dopaminergic neurons in the substantia nigra pars compacta (SNPC) (Trist et al., 2019).

Intracellular α-synuclein is cleared through the lysosomal pathway. Extracellular α-synuclein is cleared by extracellular proteolytic enzymes, or taken up by neighboring cells, especially microglia and astrocytes. Exosomes, on the other hand, represent a vehicle for egress of excess burden of the intracellular protein, potentially contributing to the transfer of α-synuclein between cells (Stefanis et al., 2019). The above methods maintain the normal homeostasis of α-synuclein.

As for other types of PD, abnormal accumulation of α-synuclein is the key pathogenesis of KRS. In patients with KRS, lysosomal function is impaired due to mutation of ATP13A2, which affects the metabolism of α-synuclein and causes the occurrence of diseases. This review discusses the findings of recent literature reports to elaborate on how ATP13A2 mutations in KRS patients cause abnormal accumulation of α-synuclein and the effects of abnormal α-synuclein accumulation in an attempt to provide new directions for future research on the pathogenesis of and therapeutic targets for ATP13A2 gene-related PD.

## Types and Effects of ATP13A2 Gene Mutations

**FIGURE 1:**
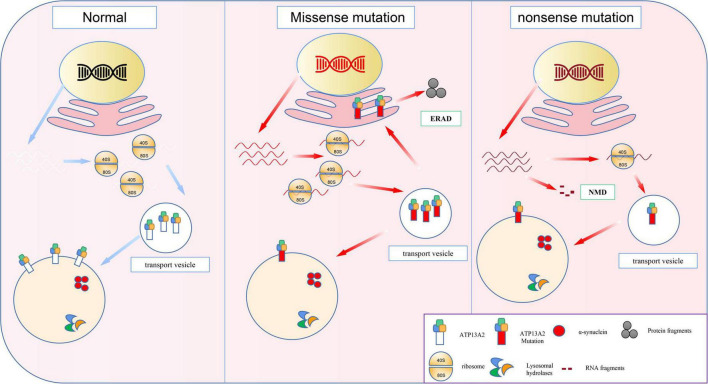
The molecular basis of loss-of-function mutations in the ATP13A2. Previous studies indicated the ATP13A2 protein is localized to the lysosomes, whereas missense mutations in the ATP13A2 gene cause retention of the protein in endoplasmic reticulum. And the mutant ATP13A2 proteins are degraded by endoplasmic reticulum-associated degradation (ERAD). Nonsense mutations produced mutant transcript and then degraded by nonsense-mediated RNA decay (NMD).

ATP13A2 gene mutations can be divided into two types, namely, frameshift mutations and base substitution mutations (Ramirez et al., 2006; Table 1). In individuals with disease-causing mutations, these mutations can be either missense mutations or nonsense mutations. Recent studies have shown that the missense mutations in the ATP13A2 gene cause the ATP13A2 protein to be misfolded, which causes the protein, which is originally localized in lysosomes, to be mislocalized to the endoplasmic reticulum (Ramirez et al., 2006; Park et al., 2011; Tan et al., 2011). The endoplasmic reticulum is one of the quality control checkpoints through which cells eliminate unwanted and potentially toxic proteins (Wan et al., 2020). After the misfolded ATP13A2 protein localizes to the endoplasmic reticulum, it activates the endoplasmic reticulum-associated degradation (ERAD) pathway and is rapidly degraded, resulting in a decrease in ATP13A2 protein levels in lysosomes (Ugolino et al., 2011; Covy et al., 2012; Matsui et al., 2013; Demirsoy et al., 2017; Figure 1). When the ATP13A2 gene mutation is a nonsense mutation, the transcribed mRNA activates the nonsense-mediated mRNA decay (NMD) pathway due to a premature termination codon (PTC). Eventually, the nonsense ATP13A2 mRNA is degraded, which also leads to a decrease in ATP13A2 protein levels in lysosomes (Hwang et al., 2010; Park et al., 2011; Figure 1). The above two degradation pathways are the molecular bases for the loss of function nature of the ATP13A2 mutations.

**TABLE 1:** Mutation types of *ATP13A2.*

Mutation types	cDNA	Protein	References
**Frameshift mutations**	c.3017_3019del	p.1006_1007del	Kara et al., 2016
	c.3057delC	p.G1019GfsX3	Ramirez et al., 2006; Kertelge et al., 2010; Weissbach et al., 2019
	c.3253delC	p.L1085WfsX4	Park et al., 2011
	c.2552_2553delTT	p.F851CfsX6	Crosiers et al., 2011
	c.1103_1104insGA	p.T368RfsX29	Paisán-Ruiz et al., 2010; Schneider et al., 2010
	c.2473delCinsAA	p.L825NfsX33	Eiberg et al., 2012
	c.1632_1653dup22	p.L552PfsX238	Williams et al., 2005; Ramirez et al., 2006
	c.2366_2367delTC	p.Leu789ArgfsX15	Pietrzak et al., 2019
	c.1422_1423del	p.P474fs	Kırımtay et al., 2021
	c.1429_1430insAAA	p.M477delinsKM	Kırımtay et al., 2021
	c.2822delG	p.S941TfsX1	Martino et al., 2015
**Base substitution mutations**	c.1535C > T	p.Thr512Ile	Estrada-Cuzcano et al., 2017
	c.2429T > G	p.Met810Arg	Bras et al., 2012
	c.1306 + 5G > A	p.G399L435del	Podhajska et al., 2012
	c.1510G > C	p.G504R	Di Fonzo et al., 2007
	c.546C > A	p.F182L	Ning et al., 2008
	c.3176T > G	pL1059R	Park et al., 2011
	c.2572C > T	p.Q825X	Malakouti-Nejad et al., 2014
	c.2629G > A	p.G877R	Santoro et al., 2011; Malandrini et al., 2013
	c.1550C > T	p.Thr517Ile	Grünewald et al., 2012
	c.364C > T	p.Gln122	Estrada-Cuzcano et al., 2017
	c.3403C > T	p.Gln1135	Estrada-Cuzcano et al., 2017
	c.1426G > T	p.A476S	Kara et al., 2016; Kırımtay et al., 2021
	c.2209C > T	p.Gln737	Pietrzak et al., 2019
	c.1330C > T	p.Arg444	Estrada-Cuzcano et al., 2017

*ATP13A2 gene mutations can be categorized as frameshift mutations and base substitution mutations, which result in two types of mutations: missense mutations and nonsense mutations.*

## Autophagy

Autophagy is an important part of the protein quality control system in cells. It can be mobilized when unnecessary protein accumulation occurs in the cell. Autophagy is responsible for the degradation of protein aggregates and damaged organelles and provides nutrients and energy for cell repair and homeostasis.

### α-Synuclein and Autophagy

α-Synuclein protein levels regulated by a balanced equilibrium between synthesis, degradation, and secretion are a major determinant of its neurotoxic potential (Vekrellis et al., 2011). In terms of protein degradation, the autophagy-lysosomal pathway (ALP) plays a huge role. Autophagy pathways include macroautophagy, chaperone-mediated autophagy (CMA), and microautophagy, each involving different mechanisms of substrate delivery to lysosome (Cerri and Blandini, 2019). Microautophagy will not be discussed further in the context of this review, because of the lack of data linking microautophagy to α-synuclein. CMA is thought to be a major mechanism for the degradation of α-synuclein in healthy cells (Cuervo, 2010). It does not involve the formation of vesicles, the substrate proteins cross the lysosomal membrane directly to the lysosomal lumen. However, this mechanism is unable to efficiently degrade oligomeric α-synuclein (Cerri and Blandini, 2019). Macroautophagy is arguably the most extensively studied autophagic proteolytic pathway. The most distinguishing feature of macroautophagy is the formation of the double-membrane bound phagophore and autophagosome (Feng et al., 2014). Furthermore, macroautophagy is mainly involved in the clearance of α-synuclein aggregates under abnormal conditions. Potential alterations in macroautophagic homeostasis process include inhibition of autophagosome formation or excess induction of the pathway, and blockade of autophagic flux usually resulting from lysosomal abnormalities that impair the fusion of autophagosomes with lysosomes (Xilouri et al., 2016). Therefore, we will mainly discuss the role of macroautophagy on the degradation of α-synuclein in this review.

### ATP13A2 and Autophagy

ATP13A2, as a lysosomal-related protein, is one of the main members of the P-type ATPase family. ATP13A2 has the same ion pump function as most members of this family (Liu et al., 2013; Chung et al., 2019). More importantly, it can also regulate the process of autophagy. Therefore, a decrease in ATP13A2 protein levels in lysosomes inevitably impairs autophagy in the cell and leads to abnormal degradation of α-synuclein.

Scholars have studied PD patients with ATP13A2 mutations and found the following changes related to lysosomal function in these patients: (I) abnormal elevation of the lysosomal pH (Vidyadhara et al., 2019), (II) increased lysosomal permeability (Rinaldi et al., 2015), and (III) impaired cathepsin maturation (Matsui et al., 2013). The abovementioned changes related to lysosomal function lead to difficulty in degrading substrates, as well as defects in the clearance of autophagic vacuoles (AVs)/autolysosomes (ALs) (Dehay et al., 2012). Large amounts of uncleared AVs/ALs accumulate in cells, further aggravating cell dysfunction (Sulzer et al., 2008) and eventually aggravating this pathological process. In addition, some studies have shown through experimental image analysis that acidic vesicles in normal cells are twice the size that of those in ATP13A2-D508N (a mutant) cells on average and have more electron compacts (Rinaldi et al., 2015). This suggests that in cells with reduced ATP13A2 protein levels, the quality of lysosomes is much lower than normal. In PD caused by mutations in the ATP13A2 gene, abnormal lysosomal function impairs autophagy, resulting in the inability of dopaminergic neurons to clear misfolded and abnormally aggregated α-synuclein, which suggests that autophagy inhibition is one of the core pathogenic processes of PD (Colasanti et al., 2014; Lopes da Fonseca and Outeiro, 2014).

Interestingly, some studies have found that although there are autophagy defects in ATP13A2-deficient mouse nerve cells, the levels of a-synuclein are normal (Kett et al., 2015). In addition, studies on SH-SY5Y cells (human neuroblastoma cells) revealed that the steady-state level and secretion of α-synuclein in ATP13A2 knockout cells are not altered. Faced with this contradictory result, Bae et al. (2014) proposed two possibilities. First, the ATP13A2 protein may play a key role in lysosomes in dopaminergic neurons and other types of cells, but at least in SH-SY5Y cells, loss of the ATP13A2 protein has a limited effect on lysosome function. Second, ATP13A2 is a member of the P5 ATPase pump family, and its function may overlap with that of other members of the family. Therefore, the expression of other P5 ATPases may compensate for the reduction in the ATP13A2 protein level to a certain extent (Bae et al., 2014).

Overall, although the current mainstream view is that autophagy impairment is the core link between the abnormal accumulation of α-synuclein and the decrease in ATP13A2 protein expression (Bento et al., 2016; Wang et al., 2019), more research is needed to explain some contradictory phenomena that have been observed.

## The Molecular Mechanism by Which ATP13A2 Regulates Autophagy

### Homeostasis of Heavy Metal Ions

**FIGURE 2:**
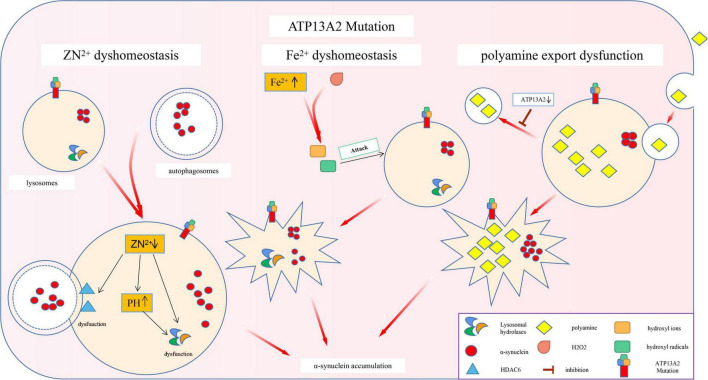
Effects of ATP13A2 gene mutation on intracellular clearance of α-synuclein. Mutations in the ATP13A2 gene lead to a decrease in ATP13A2 protein expression in lysosomes, which directly leads to the homeostasis of Zn^2+^, Fe^2+^, and other metal ions and PAs, which in turn leads to impaired autophagy and pathological accumulation of α-synuclein.

The specific substrate of P5-type ATPases has not yet been found (McKenna et al., 2020). Thus, researchers can only link ATP13A2 with metal ion transport based on the fact that most of the P-type ATPase family members are involved in metal ion transport (Palmgren and Nissen, 2011). While researching this process, scientists discovered that ATP13A2 is closely related to α-synuclein (Lopes da Fonseca and Outeiro, 2014). Specifically, ATP13A2 is a P5 ATP transport enzyme involved in cation transport and metal ion homeostasis. Mutations in this gene lead to a decrease in ATP13A2 protein levels in lysosomes, which directly leads to change in the homeostasis of Zn^2+^, Fe^2+^, and other metal ions. This leads to impaired autophagy and pathological accumulation of α-synuclein, which eventually leads to PD (Figure 2).

#### Zn^2+^

Studies have shown that loss of the ATP13A2 protein can impair the storage capacity of vesicles related to the autophagy-lysosome pathway for Zn^2+^. For example, lysosomes lacking ATP13A2 protein show lower Zn^2+^ storage capacity under physiological conditions and Zn^2+^-loading conditions, which leads to a decrease in the Zn^2+^ concentration in lysosomes (Tsunemi and Krainc, 2014; Park et al., 2015). A decrease in the concentration of Zn^2+^ mainly causes the following two changes: (I) Zn^2+^ is needed to promote the maturation of partial hydrolases in lysosomes. For example, the mature form of lysosomal acid sphingomyelinase (L-SMase), is processed from the zymogen in a Zn^2+^-dependent manner. A decrease in the Zn^2+^ concentration reduces the ability of lysosomes to hydrolyze proteins, which in turn affects the efficiency of autophagy (Tsunemi and Krainc, 2014). (II) HDAC6 is one of the molecules that control autophagosome–lysosome fusion. Zn^2+^ maintains the normal function and conformation of HDAC6 by regulating the lysosomal localization and activity of HDAC6 (Wang et al., 2019). When the concentration of Zn^2+^ decreases, the normal function of HDAC6 is impaired, thereby inhibiting autophagy.

In addition, an acidic environment is the first prerequisite for the normal function of lysosomal acid hydrolase (Mindell, 2012). It is also necessary for cathepsin maturation and activation (Kokkonen et al., 2004). Loss of the ATP13A2 protein increases the sensitivity of lysosomes to changes in the Zn^2+^ concentration; a small decrease in the Zn^2+^ concentration can cause a significant increase in the pH of the lysosome (Tsunemi and Krainc, 2014), eventually abolishing the acidic environment in the lysosome. This phenomenon has been confirmed in KRS patient cells with ATP13A2 mutations (Park et al., 2014). These phenomena lead to a decline in the function of lysosomal hydrolases and a hindrance to the autophagy process, which ultimately leads to a slowdown in the clearance of α-synuclein.

Interestingly, Park et al. (2014) have found that compared with normal cells, cells with reduced ATP13A2 protein levels exhibit changes in the expression of 13 of 19 zinc transporter (ZnT) genes, including 6 that encode ZnTs that mediate Zn^2+^ efflux (decrease cytoplasmic Zn^2+^ levels) and 7 that encode zinc import proteins (ZIP) that promote Zn^2+^ influx (increase cytoplasmic Zn^2+^ levels). Whether the changes in the expression levels of these transporters can also affect lysosomes and thereby inhibit autophagy needs to be further explored.

#### Fe^2+^

Magnetic Resonance Imaging (MRI) examination of KRS patients has revealed abnormal iron deposits in the substantia nigra, prompting researchers to study the relationship between ATP13A2 mutations and iron homeostasis imbalance (Dusek and Schneider, 2012). Because lysosomes are the main reservoirs of Fe^2+^ (Terman and Kurz, 2013), loss of the ATP13A2 protein triggers an increase in the lysosomal pH, resulting in the release of lysosomal iron into the cytoplasm (Uchiyama et al., 2008) and thus increasing the content of Fe^2+^ in the cytoplasm. This suggests that when the ATP13A2 protein level is reduced, the cell’s ability to regulate Fe^2+^ is reduced. The Fenton reaction between Fe^2+^ and hydrogen peroxide produces highly active hydroxyl radicals, which can increase lysosome membrane permeabilization (LMP) through lysosomal membrane lipid permeation (Boya and Kroemer, 2008; Johansson et al., 2010), which makes lysosomes more susceptible to damage, in turn affecting their autophagy function. Subsequent experiments have also proven that CHO cells overexpressing ATP13A2 have stronger resistance under an FeCl3-loading environment and that ATP13A2 overexpression can reduce the increase in iron deposition caused by FeCl3 treatment (Rajagopalan et al., 2016). In summary, we speculate that in the absence of the ATP13A2 protein, a large amount of Fe^2+^ is released from lysosomes into the cytoplasm, which causes lysosomal damage by affecting the integrity of the lysosomal membrane, thereby inhibiting autophagy, and causing abnormal accumulation of α-synuclein.

### Polyamine (PA) Homeostasis Imbalance

Studies have found that ATP13A2 can increase the uptake of PAs by cells, suggesting that PAs may be potential substrates of ATP13A2 (De La Hera et al., 2013). PAs, as organic cationic polymers that play an important regulatory role in the normal physiological processes of cells, participate in a variety of cell functions. However, high concentrations of PAs are cytotoxic, so their intracellular contents are strictly regulated by a variety of mechanisms (Pegg, 2016). Recent studies have shown that ATP13A2 takes up PAs (such as spermine) by promoting the cell’s own endocytic function, temporarily stores them in late endosomes/lysosomes, and then transports them to the cytoplasm. A decrease in ATP13A2 protein expression leads to accumulation of lysosomal PAs and subsequent lysosomal rupture (Van Veen et al., 2020; Vrijsen et al., 2020), which ultimately leads to impaired lysosomal function and autophagy disorders (Figure 2).

In conclusion, loss of the ATP13A2 protein would result in compromised lysosomal hydrolases and membrane integrity, ultimately impairing the clearance of α-synuclein. In addition, autophagosome-lysosome fusion is a key part of the autophagy process, and the obstruction of the fusion process will further aggravate the difficulty of α-synuclein clearance.

## Loss of ATP13A2 Function Triggers Aggregation of α-Synuclein Through Non-Autophagy Pathways

### The Secretion of α-Synuclein Is Blocked

**FIGURE 3:**
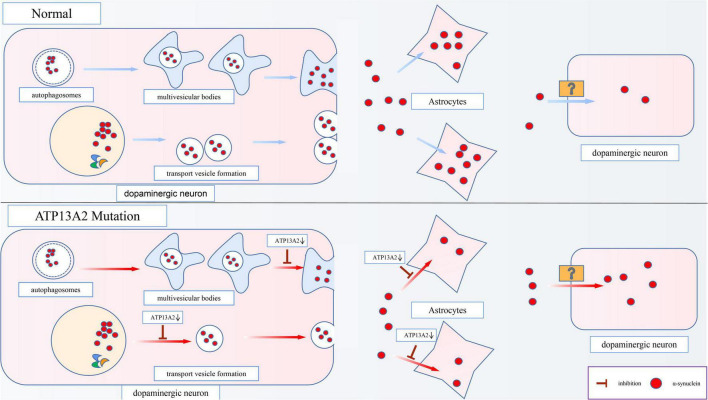
Effects of ATP13A2 gene mutation on extracellular clearance of α-synuclein. When ATP13A2 protein expression in lysosomes is reduced, lysosomal exocytosis and exosomal secretion of α-synuclein are both inhibited. In addition, a decrease in ATP13A2 protein expression in lysosomes decreases the uptake of α-synuclein by astrocytes, resulting in increased α-synuclein transfer between dopaminergic cells.

Although α-synuclein is a cytoplasmic protein mainly located at presynaptic terminals, it is also found in the extracellular space, such as cerebrospinal fluid (CSF) and plasma (Tokuda et al., 2006). The content of α-synuclein in the CSF and plasma is higher in PD patients than in normal people (El-Agnaf et al., 2006), which shows that α-synuclein has the characteristics of secreted protein. Studies have found that there are two main ways for cells to secrete α-synuclein. One is the conventional exocytosis pathway, and the other is the exosomal secretion pathway. Inhibition of these two pathways that are important for α-synuclein secretion, is an important mechanism of PD pathogenesis, and ATP13A2 also plays a pivotal role in this process (Figure 3).

#### Exocytosis

ATP13A2 can regulate conventional lysosomal exocytosis. Exocytosis is the key mechanism by which lysosomes fuse with the plasma membrane and release stored substances outside of the cell (Samie and Xu, 2014). When lysosomes undergo exocytosis, the concentration of Ca^2+^ near the lysosome surface needs to be rapidly increased. Therefore, a high Ca^2+^ concentration in the lysosome is necessary to trigger exocytosis (Xu and Ren, 2015). Tsunemi et al. (2019) have found that dopaminergic cells expressing ATP13A2 mutants (c.1550 C > T, homozygous mutation; c.3176T > G/c.3253delC, heterozygous mutation) exhibit decreased Ca^2+^ concentrations in lysosomes and increased cytoplasmic Ca^2+^ concentrations, which cause the secretion of α-synuclein to be blocked and α-synuclein accumulate in lysosomes. Interestingly, the obstruction of secretion can be reversed by agonists of TRPML1 (a lysosomal Ca^2+^ channel). Thus, researchers believe that TRPML1 may be involved in lysosomal Ca^2+^ homeostasis caused by a decrease in ATP13A2 protein levels. However, subsequent research has proven that this process is not related to TRPML1 (Tsunemi et al., 2019), suggesting that an undiscovered independent mechanism is responsible for the decrease in the Ca^2+^ concentration in lysosomes caused by the decrease of ATP13A2 protein. In general, a reduction in ATP13A2 protein expression can inhibit exocytosis of lysosomes, which leads to abnormal accumulation of α-synuclein and promotes the occurrence and development of PD.

#### Exosomal Secretion Pathway

Studies have shown that α-synuclein can also be secreted through an unconventional exosomal exocytosis pathway (Lee et al., 2005), and ATP13A2 plays a major role in the biogenesis of exosomes. Multivesicular bodies (MVBs), which are autophagosome-derived intraluminal vesicles (ILVs), can fuse with α-synuclein-containing autophagosomes to form exosomes, which are then released into the extracellular matrix after fusion with the cell membrane. However, the decrease in Zn^2+^ concentration in MVBs caused by a decrease in ATP13A2 protein expression inhibits this secretion process (Park et al., 2015). Studies show that in cells with reduced ATP13A2 protein expression, the number of ILVs in MVBs is reduced, which indicates that the release of exosomes is inhibited when ATP13A2 protein expression is reduced (Tsunemi et al., 2014). This suggests that dopaminergic cells with reduced ATP13A2 protein expression are inhibited by the secretion of α-synuclein by exosomes, which further increases the accumulation and toxicity of α-synuclein in the cell.

### Limited Extracellular Clearance of α-Synuclein

Studies using cell culture models have shown that secreted α-synuclein can be taken up by neighboring cells through cell endocytosis (Desplats et al., 2009) and that this uptake is mediated by exosomes (Danzer et al., 2012). A follow-up study in PD patients who received fetal brain tissue transplantation revealed that 11–16 years after transplantation, the content and nature of LBs in transplanted neurons were basically the same as those of LBs in dopaminergic cells in the brains of PD patients, which provides evidence for the diffusion of α-synuclein prion-like molecules (Kordower et al., 2008; Li et al., 2008; Kurowska et al., 2011). In subsequent animal experiments, it was also proven that after injection of α-synuclein into mice, the protein first spread between anatomically connected areas and then spread to lesions that were not directly connected (Luk et al., 2012). These results indicate that α-synuclein may be a prion-like protein and may be involved in the pathological process of PD through a prion-like seeding mechanism (Olanow and Brundin, 2013).

Moreover, studies have found that astrocytes, as cells that provide metabolic and nutritional support for neurons, can absorb α-synuclein from the extracellular space and degrade it (Lee et al., 2010; Loria et al., 2017). Similarly, Wakabayashi et al. (2000) have observed the accumulation of α-synuclein in the astrocytes of patients with sporadic PD. However, the accumulation ratio of α-synuclein is lower in astrocytes than in dopaminergic cells, possibly due to the higher proteolytic ability of astrocytes (Wakabayashi et al., 2000). More importantly, the latest research has revealed that a reduction in ATP13A2 protein expression decreases the uptake of α-synuclein by astrocytes, leading to increased α-synuclein transfer between dopaminergic cells (Tsunemi et al., 2020; Figure 3). Thus, we speculate that this effect may be an important mechanism by which PD lesions gradually spread from a few cells to surrounding tissues.

In conclusion, loss of the ATP13A2 protein will lead to obstacles in the exocytosis and exosome pathways, ultimately disrupting the extracellular secretion of α-synuclein. Furthermore, the restriction of the extracellular clearance process of α-synuclein would further aggravate the accumulation of α-synuclein.

## Progressive Accumulation of α-Synuclein

Studies have found that abnormal accumulation of α-synuclein can further promote abnormal α-synuclein accumulation in the cell through positive feedback loop, enhance the pathological cascade reaction, and cause continuous aggravation of disease symptoms. This may be one of the reasons for the progression of PD.

**FIGURE 4:**
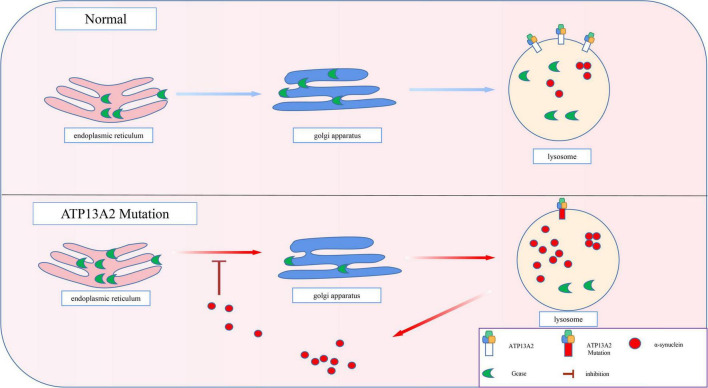
Progressive accumulation of α-synuclein. The transport of GCase is impaired by pathological accumulation of α-synuclein. Thus, GCase remains in the endoplasmic reticulum and cannot reach the lysosome to exert its normal function, which accelerates the pathological accumulation of α-synuclein.

The pathological accumulation of α-synuclein can cause vesicle transport deficits, which in turn leads to defects in endoplasmic reticulum-Golgi transport (Gosavi et al., 2002; Cooper et al., 2006). As a result, the proteins in the endoplasmic reticulum that are normally involved in the degradation of α-synuclein cannot be transported to the lysosome normally. For example, β-glucosidase (GCase), a hydrolase in lysosomes, is involved in the degradation of α-synuclein (Mazzulli et al., 2016). However, after the pathological accumulation of α-synuclein, the transport of GCase in the cell is impaired (Mazzulli et al., 2011). Thus, as shown in Figure 4, GCase remains in the endoplasmic reticulum and cannot reach the lysosome to exert its normal function (Chung et al., 2013). Animal experiments have also proven that a decrease in lysosomal GCase content can promote the accumulation of α-synuclein aggregates (Manning-Boğ et al., 2009; Rocha et al., 2015). Eventually, a positive feedback pathogenesis loop between abnormal accumulation of α-synuclein and impaired GCase transport is formed, which accelerates the pathological accumulation of α-synuclein.

## Prospects

In recent years, there have been a large number of studies on the pathogenesis of ATP13A2-related PD and explorations of therapeutic targets for this disease, and considerable progress has been made. However, much research on ATP13A2-related PD is still needed. Thus, in-depth research on the pathogenesis of ATP13A2-related PD based on basic research on PD is needed to identify therapeutic targets for delaying or even reversing ATP13A2-related PD. Moreover, in view of the fact that many PD-related genes have been proven to play an important role in a variety of diseases, exploration of the pathogenic mechanism of ATP13A2 in other diseases has gradually become a research hotspot.

First, lysosomes and mitochondria are two organelles that are essential for maintaining the stability of the intracellular environment. Their dysfunction has been closely related to many human diseases (Hutagalung and Novick, 2011; Burté et al., 2015). There have been many reports confirming the inextricable connection between mitochondria and lysosomes (Demers-Lamarche et al., 2016), and mitochondria-associated membranes (MAMs) play an important role in the process of autophagy (Sironi et al., 2020). After ATP13A2 protein expression is reduced, the concentrations of Ca^2+^ and Zn^2+^ in the cytoplasm increase. Excess Ca^2+^ enters mitochondria through Ca^2+^ channels (Cav1-L type), stimulates neuronal mitochondrial respiration and oxidative stress (Tsunemi et al., 2019), and impairs mitochondrial function by producing additional oxidation products. Increased Zn^2+^ concentration in the cytoplasm increase the accumulation of Zn^2+^ in mitochondria and increases the production of mitochondrial ROS (Dineley et al., 2005), which leads to a decrease of MMP and an increase in the production of ROS, causing cell dysfunction and death (Park et al., 2014). In addition, a reduction in ATP13A2 protein expression will lead to a decrease in the concentrations of cytoplasmic PAs, which cannot effectively reduce mitochondrial-derived ROS and affect mitochondrial function (Vrijsen et al., 2020). Thus, we speculate that a reduction in ATP13A2 protein expression can inhibit autophagy by affecting mitochondrial function, which in turn leads to abnormal accumulation of α-synuclein; however, subsequent experiments are needed to prove this hypothesis.

Second, when ATP13A2 protein expression is reduced, the cation concentration in the lysosome as well as the lipid content in the lysosome changes. Animal studies have shown that mice with mutations in the ATP13A2 gene have a significant increase in the contents of lipids, especially Bis (monoacylglycerol) phosphate (BMP), which abnormally accumulates, in the lysosome (Kett et al., 2015). BMP is a lipid that is present in late endosomes and lysosomes (Gallala and Sandhoff, 2011) and plays an important role in lipid degradation in acidic vesicles and exosomal biosynthesis (Marcos et al., 2019). For example, the formation of ILVs requires BMP (Bissig and Gruenberg, 2013). Due to the acidic environment of lysosomes, the negative charge of BMP is the key to its function, and a decrease in ATP13A2 protein expression can affect the pH of lysosomes. Thus, we speculate that ATP13A2 can alter the normal function of BMP by affecting the pH of the lysosome. Lysosomes contain a variety of lipids, and these lipids may also be affected by the pH of the lysosome, which changes the physiological functions of the lysosome. The lipid digestion ability of the lysosomal membrane is changed or lipids are redistributed, which disrupts the integrity of the lysosomal membrane and ultimately causes autophagy inhibition and abnormal accumulation of α-synuclein. However, further research is needed on these processes.

Third, the latest research shows that the P5-type ATPase CATP-8 is located in the endoplasmic reticulum. It can remove misplaced mitochondrial tail-anchored (TA) and signal-anchored (SA) proteins from the endoplasmic reticulum through non-ERAD processes (Qin et al., 2020). In addition, a P5-type ATPase located in the endoplasmic reticulum (ATP13A1) can directly interact with TA proteins to clear these proteins (McKenna et al., 2020). Elimination of mislocalized proteins by P5 ATPases helps maintain the dynamic balance of the endoplasmic reticulum and mitochondria and may represent a previously undiscovered cell protection and quality control mechanism. As a member of the P5 ATPase family, the ATP13A2 gene can encode the protein Atp13a2Isoform-3, which is located in the endoplasmic reticulum. However, the function of this protein is unknown (Ugolino et al., 2011). Whether this protein has the same clearance function, whether it affects cell function by altering the dynamic balance of endoplasmic reticulum and mitochondria, and whether it regulates the degradation of α-synuclein needs to be further studied.

Fourth, it is interesting that although α-synuclein is highly enriched in the nervous system, it is not limited to nerve tissue. A -synuclein has been detected in muscle, the kidney, the liver, the lungs, the heart, the testis, blood vessels, the CSF, the plasma, platelets, lymphocytes, and red blood cells (Uéda et al., 1993; Jakes et al., 1994; Hashimoto et al., 1997; Askanas et al., 2000; Shin et al., 2000; Li et al., 2002; Tamo et al., 2002; Ltic et al., 2004; Schultheis et al., 2004; Nakai et al., 2007; Burre et al., 2018). Why then do mutations in the ATP13A2 gene only cause excessive accumulation of the protein in dopaminergic neurons and produce such serious consequences? Is there a unique way to clear α-synuclein in other tissues and cells to prevent the abnormal accumulation of α-synuclein? These questions are worthy of in-depth research, which may provide a new direction for the clinical treatment of PD.

Finally, autophagy has been shown to play an important role in tumorigenesis and development. PD-related genes such as PARK2 (Parkin) and PARK6 (PINK1) have been proven to be involved in the occurrence and development of tumors (Denison et al., 2003; Quinsay et al., 2010; Veeriah et al., 2010; Shah et al., 2012; Pugh et al., 2013). For example, in the field of colorectal cancer, studies have found that among 100 cases of colorectal cancer tissues, 33 cases were associated with PARK2 (Poulogiannis et al., 2010). The latest study found that ATP13A2 can reduce tumorigenesis by blocking autophagic flux in colon cancer (Chen et al., 2020). This suggests that ATP13A2 may also be an important gene for tumorigenesis and development and is worthy of further study. Furthermore, autophagy is critically related to the abnormal accumulation of α-synuclein, and studies have shown that ATP13A2 can inhibit the toxicity of α-synuclein (Khurana et al., 2017). Thus, is there a connection between a-synuclein and tumors? Is the occurrence of colon cancer related to the toxicity of α-synuclein? Can α-synuclein be used as a prognostic marker for colon cancer? These questions are worthy of further exploration.

## Author Contributions

FZ and ZW conceived, designed, and drafted the manuscript. FL wrote the original draft preparation. JT, NG, and XL contributed to the review and editing of the manuscript. CL contributed to the language modification and guidance. All authors read and approved the final manuscript.

## Conflict of Interest

The authors declare that the research was conducted in the absence of any commercial or financial relationships that could be construed as a potential conflict of interest.

## Publisher’s Note

All claims expressed in this article are solely those of the authors and do not necessarily represent those of their affiliated organizations, or those of the publisher, the editors and the reviewers. Any product that may be evaluated in this article, or claim that may be made by its manufacturer, is not guaranteed or endorsed by the publisher.
